# Aberrant tumor vasculature. Facts and pitfalls

**DOI:** 10.3389/fphar.2024.1384721

**Published:** 2024-03-21

**Authors:** Domenico Ribatti

**Affiliations:** Department of Translational Biomedicine and Neuroscience, University of Bari Medical School, Bari, Italy

**Keywords:** angiocrine factors, angiogenesis, endothelial cells, tumor growth, resistance

## Abstract

Endothelial cells form a single cell layer lining the inner walls of blood vessels and play critical roles in organ homeostasis and disease progression. Specifically, tumor endothelial cells are heterogenous, and highly permeable, because of specific interactions with the tumor tissue environment and through soluble factors and cell–cell interactions. This review article aims to analyze different aspects of endothelial cell heterogeneity in tumor vasculature, with particular emphasis on vascular normalization, vascular permeability, metabolism, endothelial-to-mesenchymal transition, resistance to therapy, and the interplay between endothelial cells and the immune system.

## Introduction

The endothelium of large and small vessels, including arteries characterized by continuous endothelium, aligned in the direction of flow and without valves, veins, characterized by continuous endothelium, not aligned in the direction of flow, with valves, and capillaries, characterzied by endothelium adapted to the underlying tissues and with phenotypic differences between different vascular beds (Ribatti et al., 2002; Crivellato et al., 2007). Genetic and environmental factors influence endothelial heterogeneity through the release of specific soluble factors or cell–cell interactions, involved in determining specific vascular structure and function (Ribatti et al., 2002; Crivellato et al., 2007). High permeability and fenestrations are dependent on the secretion of vascular permeability factor/vascular endothelial growth factor (VPF/VEGF) (Dvorak et al., 1992; Esser et al., 1998). Capillary endothelial cells show heterogenic characteristics between different organs (continuous thick capillaries are present in skeletal muscle, cardiac smooth muscle, and testes; thin continuous capillaries are present in the central nervous system and dermis; sinusoids are present in the liver, spleen, and bone marrow; fenestrated capillaries are present in endocrine glands), and also in single organs, such as in the kidney where are present fenestrated endothelial cells in peritubular capillaries, discontinuous endothelial cells in glomerular capillaries, and continuous endothelial cells in other regions (Ribatti et al., 2009). In both between-organ and between-vessel type differences, heterogeneity arises from the necessity for endothelial specialization. Endothelial cells in these differing vascular beds have unique molecular functions that drive their particular structure and molecular phenotype.

Endothelial cells are also able to secrete specific angiocrine factors, such as VEGF, angiopoietin-2 (Ang-2), bone morphogenetic protein-2, -4 (BMP-2, -4), C-X-C motif chemokine-12 (CXCL-12), fibroblast growth factor-2 (FGF-2), granulocyte colony-stimulating factor (G-CSF), granulocyte-macrophage colony-stimulating factor (GM-CSF), insulin growth factor-1 (IGF-1), interleukin-3, -6, -8 (IL-3, IL-6, IL-8), pentraxin-3 (PTX-3), placental growth factor (PlGF), platelet-derived growth factor (PDGF), transforming growth factor beta (TGF-β), able to modulate the growth and morphogenesis of specific organs, such as the liver, kidney, and bone marrow (Ribatti et al., 2023a; b, c), and are involved also in cancer progression and metastasis (Maishi et al., 2019; Butler et al., 2010).

A remarkable endothelial cell heterogeneity in different organs has been demonstrated by single-cell RNA sequencing (scRNA-seq) (Kalucha et al., 2020), which is altered in tumors, such as occurs in breast cancer, where subsets of tumor endothelial cells are involved in cancer metabolism and transport (Geldhof et al., 2022). Moreover, endothelial cells from lung cancer compared to normal endothelial cells show a strong signature in signaling pathways such as the MYC and PI3K/Akt/mTOR (Lambrechts et al., 2018). Pericytes in tumor vessels are present but abnormal, lacking an intimate association with endothelial cells (Morikawa et al., 2002), and VEGF inhibitors induce a close association of pericytes with endothelial cells (Inoi et al., 2004).

This review article aims to analyze different aspects of endothelial cell heterogeneity in tumor vasculature, with particular emphasis on vascular normalization, vascular permeability, metabolism, endothelial-to-mesenchymal transition, resistance to therapy, and the interplay between endothelial cells and the immune system.

## Tumor endothelial cells

Tumor endothelial cells are characterized by immaturity and leaky, lack of perivascular cell coverage, and loss of basement membrane (Baluk et al., 2003; di Tomaso et al., 2005) favoring the passage of T cells (Di Russo et al., 2017), form arteriovenous shunts (a category of vessels with a very low resistance to flow), show chaotic and sluggish blood flow, which does not follow a unidirectional path, and proceed in alternating directions (Chaplin et al., 1987; Mc Donald and Baluk, 2002; Mc Donald and Choyke, 2003). Only 0.1%–3% of all normal endothelial cells turn over daily declining with age (Schwartz and Benditt, 1973), whereas in tumors, endothelial cell turnover may be 20–2,000 times the rate in normal tissues (Hobson and Denekamp, 1984). Vessels are more numerous at the tumor-host interface, whereas the internal portions are less vascularized. Glomeruloid microvascular proliferations, consisting of poorly organized structures resembling renal glomeruli, have been found in different human tumors (Straume et al., 2003). Alternative vascularization mechanisms, different from classic angiogenesis, have been described in tumors, including vascular co-option, vasculogenic mimicry, and intussusceptive microvascular growth (Ribatti and Pezzella, 2021).

Tumor endothelial cells are genetically unstable with embryonic characteristics promoting pro-tumor and anti-inflammatory behavior (St Croix et al., 2000; Seaman et al., 2007; Huijbers et al., 2022). The gene expression patterns of vascular endothelial cells derived from normal and malignant colorectal tissues have been investigated (St Croix et al., 2000), showing that among 79 transcripts differentially expressed, 46 were elevated and 33 were expressed at lower levels in tumor-associated endothelial cells. The transcriptional profiling results of tumor endothelial cells from multiple studies and multiple tumor types have been compared (Aird, 2009), showing that a few overexpressed genes were shared by different tumors including matrix metalloproteinase 9 (MMP9) (ovary and breast), HEYL (breast and colon), and secreted protein acidic and rich in cysteine (SPARC) (breast and colon and brain), whereas most genes were limited to one tumor type or invasive tumors. The upregulation of several genes, such as lysyl oxidase (Osawa et al., 2013), suprabasin (Alam et al., 2014), and biglycan (Yamamoto et al., 2012) enhances the migration and tube-forming capacity of tumor endothelial cells. Upregulated expression of stemness genes such as stem cell antigen-1 (Sca-1) and MDR-1 (Matsuda et al., 2010) and aldehyde dehydrogenase (ALDH) (Ohmura-Kakutani et al., 2014) has been demonstrated in tumor endothelial cells, as a part of the tumor endothelial cell population (Nagy and Dvorak, 2012; Goveia et al., 2020). CD133^+^ tumor endothelial cells have a higher frequency of aneuploidy than the CD133^-^ ones, suggesting that tumor endothelial cells originating from progenitor cells are involved in inducing genetic instability in these cells (Akino et al., 2010). Progenitor-derived tumor endothelial cells that express CD133 are undifferentiated, highly proliferative cells (Rafii et al., 2002).

In tumor endothelial cells proangiogenic molecules, including VEGF receptor (VEGFR)-1, −2, −3, VEGF-D, Tie-2, and Ang-1 are upregulated when compared with normal endothelial cells (Bussolati et al., 2003), favoring a proangiogenic phenotype (Matsuda et al., 2010). Moreover, tumor endothelial cells show different responsiveness to epidermal growth factor (EGF) (Amin et al., 2006), adrenomedullin (Tsuchiya et al., 2010), and VEGF (Matsuda et al., 2010) compared with normal endothelial cells.

Endothelial cells from high metastatic tumors show upregulation of VEGF, VEGFR-1, VEGFR-2, MMP-2, MMP-9, and display increased Akt phosphorylation compared with low metastatic ones (Ohga et al., 2012).

Activin-like receptor kinase 1 (ALK1) expression in tumor endothelial cells is a prognostic factor for metastasis of breast cancer, because pharmacologic targeting of ALK1 provided long-term therapeutic benefit in mouse models of mammary carcinoma, accompanied by strikingly reduced metastatic colonization (Cunha et al., 2015). Prolyl hydroxylase domain protein 2 (PHD2) deficiency normalized tumor blood vessels, associated with a reduction of tumor cell intravasation and metastasis (Mazzone et al., 2009). Biglycan is upregulated in tumor endothelial cells of metastatic tumors, facilitating the migration of toll-like receptor 2/4^+^tumor cells, which increases circulating tumor cells and lung metastasis (Maishi et al., 2016).

## Tumor endothelial cells and vessel normalization

Vessel normalization through anti-VEGF agents improves perfusion and more efficient local delivery of oxygen, decreases vascular leakiness, and reduces intratumoral hypoxia improving pericyte recruitment (Jain, 2001; Huang et al., 2012) allowing drug delivery and immune cell infiltration, and increasing the sensitivity of the tumor cells to radiation and chemotherapy.

VEGFR2 blockade induces upregulation of Ang1 which promotes endothelial cell junctions thickening and stabilization of endothelial cells (Winkler et al., 2004). Moreover, VEGF blockade resulted in reduced interstitial fluid pressure, and tissue edema, increased perfusion, and enhanced oxygenation and drug delivery to the tumor core. The transient effect of tumor vascular normalization might be associated either with excessively high and continuous administration of anti-angiogenic drugs or the development of drug resistance due to the activation of other pro-angiogenic factors (Bergers and Hanahan, 2008).

## Tumor endothelial cells and vascular permeability

Plasma components extravasate across vascular endothelium by paracellular (through inter-endothelial cell junctions) and transcellular [caveolae, fenestrae and vesiculo-vacuolar organelles (VVOs)] routes. Tumor vessel leakiness occurs through VVOs and trans endothelial cell pores resulting from VVOs activated by VEGF (Feng et al., 1999; Ribatti and Tamma, 2018).

Although tumor vessels can have barrier defects large enough for hemorrhage, plasma leakage in tumors is limited by reduced driving force due to poor vascular perfusion and high interstitial pressure resulting from impaired lymphatic drainage (Mc Donald and Baluk, 2002; Jain et al., 2014.) High tumor interstitial fluid pressure causes blood vessel collapse and impedes blood flow, and delivery of therapeutics to the central region of the tumor, causing hypoxia in tumor tissue (Boucher and Jain, 1992). Hypoxia, in turn, makes tumor cells resistant to radiation therapy, induces numerous genes that make tumor cells resilient to cytotoxic drugs, causes genetic instability within tumor cells, and triggers genetic mutations making the tumor cells more malignant and prone to metastasis. Low permeability tumors may overexpress Ang-1 and under express VEGF or PlGF, whereas those with high permeability may lack Ang-1 or overexpress its antagonist Ang-2 (Jain and Munn, 2000). Hypoxia and the secretion of angiogenic cytokines, favor tumor revascularization through the mobilization of bone marrow-derived endothelial progenitor cells (Gao et al., 2009).

## Tumor endothelial cells and metabolism

Emerging evidence has suggested that endothelial metabolism allows endothelial cells to adapt to the tissue-specific functions are supply the tissue with the necessary nutrients that it imports from the circulating blood. Dysregulation of endothelial cell metabolism has been associated with many diseases including atherosclerosis, diabetes, neovascular eye disease, and cancer. Glycolysis-related genes are overexpressed in the transcriptomic signature of tumor endothelial cells (Rohlenova et al., 2020). Glucose uptake and glycolysis are higher in tumor endothelial cells (Garcia-Caballero et al., 2022). Dysfunctional metabolism in tumor endothelial cells produces excessive lactate and 2-hydroxyglutarate (2-HG), inhibiting the cytotoxic functions of T cells (Tyrakis et al., 2016). Altered glycolysis in tumor endothelial cells due to an upregulated expression of glycolysis genes, contributes to structural deformities observed in tumor blood vessels (Cantelmo et al., 2016). Tumor endothelial cells proliferate under lactic acidosis caused by tumor cell glycolytic metabolism, and the pH regulator, carbonic anhydrase 2 (CAII), is involved in resistance to low pH in tumor endothelial cells (Annan et al., 2020).

## Tumor endothelial cells and endothelial to mesenchymal transition

Endothelial cells may de-differentiate into mesenchymal stem-like cells (Medici and Kalluri, 2012), a process named endothelial to mesenchymal transition (EndoMT) (Ribatti, 2022). During EndoMT, endothelial cells lose endothelial markers, including platelet endothelial cell adhesion molecule-1 (PECAM-1), Tie-2, and vascular endothelial (VE)-cadherin, and acquire mesenchymal markers, including N-cadherin, fibroblast specific protein-1 (FSP-1), alpha-smooth muscle actin (αSMA), types I/III collagen, and vimentin. The endothelial cytoskeleton rearrangement associated with EndoMT promotes intravasation and extravasation of tumor endothelial cells (Reymond et al., 2013).

Tumor-induced EndoMT is associated with the activation of pro-inflammatory pathways in endothelial cells (Nie et al., 2014). Endothelial cells undergoing tumor induced EndoMT express higher levels of the VEGF gene (Hong et al., 2018), and EndoMT contributes to metastatic extravasation and intravasation (Dudley et al., 2012). In glioblastoma, tumor endothelial cells secrete extracellular vesicles which induce mesenchymal reprogramming of cancer cells (Adnani et al., 2002).

## Tumor endothelial cells and resistance to therapy

Renal carcinoma endothelial cells are resistant to vincristine (Bussolati et al., 2003), and hepatocellular carcinoma endothelial cells are resistant to 5-fluorouracil and Adriamycin (Xiong et al., 2009; Ohga et al., 2012). Endothelial cells of metastatic melanoma have a higher expression of MDR-1 (Akiyama et al., 2012) and ALDH and are resistant to paclitaxel (Hida et al., 2017). IGFBP7 expressed by tumor endothelial cells suppresses IGF1R signaling and the stem-cell-like property of tumor cells. Chemotherapy triggers tumor endothelial cells to suppress IGFBP7, and the upregulation of IGF1 activates the FGF4-FGFR1-ETS2 pathway and accelerates the conversion of tumor cells to chemo-resistant tumor stem-like cells (Cao et al., 2017).

Vasculogenic mimicry and vascular co-option are involved in intrinsic and acquired resistance. Vasculogenic mimicry is associated with poor prognosis, reduced survival, and a high risk of cancer recurrence (Li et al., 2016). Histological examination of glioma bioptic specimens of patients who died after receiving treatment with cediranib, an inhibitor of VEGFR-2 (di Tomaso et al., 2011), or bevacizumab (de Groot et al., 2010) demonstrated that glioma cells grow around pre-existing vessels in a non-angiogenic fashion.

## Tumor endothelial cells and the immune system

Tumor endothelial cells promote the loss of protective anti-cancer immunity, the so-called “endothelial anergy” (De Sanctis et al., 2018), corresponding to the unresponsiveness of tumor endothelial cells to pro-inflammatory stimulation, impeding the adhesion and migration of immune cells (Griffioen et al., 1996; Lambrechts et al., 2018). Endothelial anergy is reversible (several therapeutic approaches have been developed to reverse tumor endothelial cell anergy and thus favor the intra-tumoral recruitment of anti-tumor immune cells) and may be used as a therapeutic strategy, suggesting that blocking this mechanism in tumor endothelial cells favors the influx of immune cells (Facciabene et al., 2017). It has been demonstrated that anti-angiogenic therapy could revert endothelial cell anergy, allow leukocytes to infiltrate tumors, and stimulate anti-tumor immunity (Nowak-Sliwinska et al., 2023).

Endothelial cells regulate leukocyte extravasation through the expression of adhesion molecules, such as selectins, intercellular cellular adhesion molecule-1 (ICAM-1), vascular cellular adhesion molecule-1 (V-CAM-1), PECAM-1, and through the weakening of endothelial cell-cell contacts allowing transmigration of immune cells (Wettschurek et al., 2019). Tumor endothelial cells fail to express proper ICAM-1 and VCAM-1 levels (Huijbers et al., 2022). The glycosylation of surface molecules modulates the adhesive properties of tumor endothelial cells and can either enhance or reduce immune cell migration (Chandler et al., 2019). The vasoconstrictive peptide endothelin 1 (ET1) is associated with ICAM-1 expression and the decreased presence of tumor-infiltrating leukocytes (Buckanovich et al., 2008).

Tumor endothelial cells secrete IL-6 and CSF-1 which promote anti-tumor alternative macrophage polarization by triggering Akt1/mTOR pathway, resulting in anti-inflammatory and pro-tumorigenic macrophage activation (Wang et al., 2018). Endothelial cell-derived CXCL-12 promotes monocyte recruitment and macrophage education by tumor cells (Alsina-Sanchis et al., 2022). Endothelial cell-derived PGE2 and IL-10 restrict T-cell activity (Mulligan and Young, 2010).

Tumor endothelial cells downregulate genes responsible for major histocompatibility complex (MHC) expression impeding their antigen-presenting functions, thus contributing to tumor immune evasion (Goveia et al., 2020). The binding of inhibitory immune checkpoints (e.g., PD-1) on CD8^+^ cells with their ligands (e.g., PD-L1 and PD-L2) on tumor endothelial cells inhibits T cell activation and these ligands can be upregulated by tumor endothelial cells on proinflammatory factors (Georganaki et al., 2018).

## Discussion

In this review, we addressed the abnormality and heterogeneity of tumor endothelial cells through the analysis of different aspects of this heterogeneity, including vascular normalization, vascular permeability, metabolism, endothelial-to-mesenchymal transition, resistance to therapy, and the interplay between endothelial cells and the immune system.

Endothelial cell heterogeneity can be quantified through epigenomic, transcriptomic, and proteomic studies. The characterization and understanding of endothelial cell heterogeneity have advanced in the past years, due to the development of single-cell OMICs approaches. ScRNA-seq methods for tissue-derived cell suspensions and cultured cell populations have been an area of intense development. Single-cell transcriptional sequencing (scRNA-seq) techniques enable gene expression analyses at a single cell level, investigating the transcriptional output of cells in both normal and tumoral tissue samples. Thanks to the identification of preferentially expressed genes, gene expression study permits to identification of not only different cell types, but also various cell states progression along the cell cycle, different metabolic states, or rather the diversity within each of the clusters defined as “cell types.” More work on vascular single-cell analysis is required to establish the principles of endothelial activation and their interpretation for the different tissue challenges that require vascular adaptations (Pasut et al., 2021; Becker et al., 2023).

The knowledge on tumor endothelial cell phenotypes is under continuous development, even if their role in immune escape and the response to immune and anti-angiogenic therapies should be further analyzed and clarified. Notably, most human tumor types contain varying numbers but only a small population of angiogenic tumor endothelial cells, the targets of anti-angiogenic therapies, contributing to the limited efficacy of and resistance to these therapies.
